# Oral Microbiota and Risk for Esophageal Squamous Cell Carcinoma in a High-Risk Area of China

**DOI:** 10.1371/journal.pone.0143603

**Published:** 2015-12-07

**Authors:** Xingdong Chen, Björn Winckler, Ming Lu, Hongwei Cheng, Ziyu Yuan, Yajun Yang, Li Jin, Weimin Ye

**Affiliations:** 1 Department of Medical Epidemiology and Biostatistics, Karolinska Institutet, Stockholm, Sweden; 2 Ministry of Education Key Laboratory of Contemporary Anthropology and State Key Laboratory of Genetic Engineering, Collaborative Innovation Center for Genetics and Development, School of Life Sciences, Fudan University, Shanghai, China; 3 Fudan-Taizhou Institute of Health Sciences, Taizhou, China; 4 Clinical Epidemiology Unit, Qilu Hospital of Shandong University, Jinan, China; 5 Taixing People’s Hospital, Taixing, China; Duke Cancer Institute, UNITED STATES

## Abstract

Poor oral health has been linked with an increased risk of esophageal squamous cell carcinoma (ESCC). We investigated whether alteration of oral microbiota is associated with ESCC risk. Fasting saliva samples were collected from 87 incident and histopathologicallly diagnosed ESCC cases, 63 subjects with dysplasia and 85 healthy controls. All subjects were also interviewed with a questionnaire. V3–V4 region of 16S rRNA was amplified and sequenced by 454-pyrosequencing platform. Carriage of each genus was compared by means of multivariate-adjusted odds ratios derived from logistic regression model. Relative abundance was compared using Metastats method. Beta diversity was estimated using Unifrac and weighted Unifrac distances. Principal coordinate analysis (PCoA) was applied to ordinate dissimilarity matrices. Multinomial logistic regression was used to compare the coordinates between different groups. ESCC subjects had an overall decreased microbial diversity compared to control and dysplasia subjects (*P*<0.001). Decreased carriage of genera *Lautropia*, *Bulleidia*, *Catonella*, *Corynebacterium*, *Moryella*, *Peptococcus and Cardiobacterium* were found in ESCC subjects compared to non-ESCC subjects. Multinomial logistic regression analyses on PCoA coordinates also revealed that ESCC subjects had significantly different levels for several coordinates compared to non-ESCC subjects. In conclusion, we observed a correlation between altered salivary bacterial microbiota and ESCC risk. The results of our study on the saliva microbiome are of particular interest as it reflects the shift in microbial communities. Further studies are warranted to verify this finding, and if being verified, to explore the underlying mechanisms.

## Introduction

The positive association between alcohol use, tobacco smoking and the risk of esophageal squamous cell carcinoma (ESCC) has been well established, especially in Western countries. However, in areas with high incidence of ESCC, including the so-called “Asian esophageal caner belt”, the major factors contributing to ESCC are yet to be established.[1] Recently, an association between indicators of poor oral hygiene and ESCC has been reported in studies from several high-risk areas of China,[2] India,[3] Iran[4], and from other areas including Latin America[5] and Japan.[6] Furthermore, poor oral health was reported as a risk factor for the precursor lesion of ESCC, *i*.*e*. esophageal squamous dysplasia,[7] and it may act synergistically in increasing the risk of ESCC with other risk factor (*e*.*g*. gastric atrophy).[8] There is reason to assume that poor oral health and hygiene are critical risk factors for ESCC in high-risk areas.

The underlying mechanism for the associations between oral health status and ESCC risk is not completely understood. It is well established that the oral microbiome plays a critical role in the maintenance of a normal oral physiological environment and in development of oral diseases, including periodontal diseases and tooth loss. Although little studied, the oral microbiome may be important in cancer and other chronic diseases, through direct metabolism of chemical carcinogens (e.g. nitrite, ethanol) [9, 10] and through systemic inflammatory effects [11]. We assumed that a stronger underlying association of ESCC risk with oral microbiome profiles would exsit. Although some specific bacterial species in tissue and saliva have been linked to an elevated risk of ESCC by targeted approach,[12, 13] to date few studies have systemically investigated the relation between oral microbiota and ESCC risk. In current study, we aim to investigate the potential association between oral microbiota in saliva and ESCC risk using 16S rRNA amplicon sequencing approach, based on a large case-control study conducted in Taixing, an area with a high incidence of ESCC.

## Materials and Methods

### Study base

A case-control study on esophageal cancer was conducted during October of 2010 and March of 2012 in Taixing of Jiangsu Province, China. Briefly, cases were recruited mainly from endoscopy units at the four largest hospitals of Taixing (the People's Hospital of Taixing, the Second People's Hospital of Taixing, the Third People's Hospital of Taixing and the Hospital of Traditional Chinese Medicine of Taixing). More than 90% of the patients in this area are referred to these hospitals. Subjects who were suspected to have esophageal cancer under endoscopy were asked to participate in the study. Case recruitment was also supplemented by additional linkage to the local Cancer Registry, and sample collection of the supplementary cases was conducted at the end of the same year. Control subjects, frequency matched to the cases of ESCC on sex and age in 5-year groups and randomly selected from the Taixing population register, were enrolled into the study during the same period with cases. All subjects in the study were restricted to local inhabitants who have lived in Taixing for at least 5 years prior to diagnosis date for cases or interview date for controls.

The current study is a sub-project of the case-control study focusing on the relation between oral microbiota and ESCC risk. Cases were those who were recruited from endoscopy room during the period from October of 2010 to August of 2011 (N = 331), and controls were those who were recruited during June of 2011 and August of 2011 (N = 400). In order to avoid possible confounding which might affect diversity of oral microbiota, *i*.*e*. ambient temperatures and dietary habits in different seasons, cases collected during November of 2010 and March of 2011 were excluded (N = 124). Cases without histopathological confirmation, complete questionnaire or saliva sample were also excluded (N = 36). The study base of the current study thus included 171 ESCC cases and 400 controls. Since saliva collection was performed after endoscopy for ESCC cases, while for control subjects it was performed only after fasting overnight, we could not exclude the possibility of contamination by endoscopy which would affect the diversity of oral microbiota. Therefore, we included 80 subjects with a suspected diagnosis of esophageal dysplasia who also underwent endoscopy during the whole study period as another “control” group in the current study.

### Data collection by interview

All subjects underwent face-to-face interviews by trained interviewers using a standardized questionnaire. The questionnaire covered detailed information on age, sex, education, smoking, alcohol drinking, family history of ESCC and other potential confounders of interest. Dietary habits 10 years before interview were collected using a food frequency questionnaire specifically designed for this population.[14] The trained personnel counted each subject’s number of teeth, recorded the number of missing and filled teeth (the sum of which was the MFT score) [4] and oral hygiene habits (times of tooth brushing per day).

### Saliva DNA extraction and subject selection

About 2~3 mL of saliva was collected from each participant after overnight fasting. Saliva collection was before antitumor treatment of cases, and for both cases and controls, no prescription of antibiotics one month before interview was reported. Saliva sample was mixed with 3mL lysis buffer (50mM Tris, pH 8.0, 50mM EDTA, 50mM sucrose, 100mM NaCl, and 1% sodium dodecyl sulfate). The mixture was delivered to the laboratory same day of collection and stored in the -20°C freezers. A modified high-salt DNA extraction method was used to extract DNA from saliva samples. Thirty microliters of proteinase K (20mg/mL, Sigma) and 150uL of 10% sodium dodecyl sulfate were added to 2mL of the mixture, which was then incubated overnight at 53°C in a shaking water bath. After addition of 400uL of 5M NaCl and incubation for 10 min on ice, the mixture was distributed equally into 2-mL centrifuge tubes and centrifuged for 10min at 13,000 rpm in an Eppendorf 5415D centrifuge. The supernatant from each tube was transferred to a new tube to which 800uL of isopropanol was added; the tubes were then incubated 10 min at room temperature and centrifuged for 15min at 13,000 rpm. The supernatants were discarded, and the sediments were washed twice with 500uL of 70% ethanol; then the sediments were dried and dissolved in 30uL of double-distilled water. DNA concentration of each sample was measured by the NanoDrop spectrophotometer.

We first selected study subjects according to the DNA quality standards (DNA concentration: ≥20ng/uL; A260/280: 1.8~2.0; total amount: ≥400ng) set by the BGI Company (Shenzhen, China) which conducted sequencing for current study. Eventually, 100 of 171 ESCC cases, 70 of 80 dysplasia controls and 312 of 400 controls met the standards. We thus enrolled 100 ESCC cases and 70 dysplasia control subjects, and for healthy controls we selected 100 controls frequency matched to the ESCC cases by sex and age in 5-year groups. Meanwhile, pathological sections were re-reviewed by an experienced pathologist, and one case from ESCC group was re-diagnosed as esophageal adenocarcinoma (excluded), three subjects from the dysplasia group were re-diagnosed as ESCC (regrouped into ESCC). Finally, 102 ESCC cases (ESCC group), 67 dysplasia control subjects (Dysplasia group) and 100 healthy controls (control group) were included in the current study.

### Sequencing, data processing and statistical analysis

16S ribosomal RNA (16S rRNA) amplicons covering hypervariable regions V3 to V4 were generated using primers (341_F- CCTACGGGNGGCWGCAG and 805_R- CTACCRGGGTATCTAATCC) incorporating Roche 454 FLX Titanium adapters (Branford, CT) and sample barcode sequences.[15] Amplicons were sequenced using single-read sequencing method following the manufacturer’s specifications on the 454 Roche FLX Titanium pyrosequencing platform. Laboratory personnel were blinded to the case–control status. All the procedures except DNA extraction were conducted by the BGI Company.

Amplicon reads with mismatches in either primer or barcode were discarded and the remaining reads were stripped of barcode and primers. The *fastq_filter* command of USEARCH 7.0.1001[16] was used to discard reads with more than one expected error as well as to truncate reads to a length of 300 nucleotides. Shorter reads were discarded. The quality-filtered reads were abundance sorted and clustered into operational taxonomic units (OTUs) using the USEARCH *cluster_otus* command with 97% sequence identity. Singleton reads were ignored in the *cluster_otus* command to avoid spurious OTUs. Chimera removal was performed as part of the OTU clustering step and by using the USEARCH *uchime_ref* command against the “Gold” ChimeraSlayer reference database (r20110519).[17] Abundance tables were created by aligning the quality-filtered reads against the database of OTUs with the *usearch_global* command.

QIIME 1.7.0[18] was used to assign taxonomy and to generate a phylogenetic tree after aligning the reads and filtering alignments. The scripts used in this step were: *assign_taxonomy_rdp*.*py* to assign taxonomy against the Greengenes database (v12_10)[19] with the RDP classifier (v2.2), *make_phylogeny*.*py* to build a phylogenetic tree using FastTree (v2.1.3),[20] *align_seqs_pynast*.*py* to align with PyNAST[21] against the Greengenes core reference alignment, and *filter_alignment*.*py* to filter the PyNAST alignment.

Data analysis and visualization was performed using R (v3.0.1) and the package phyloseq (v1.4.5).[22] Samples with less than 1000 depth were discarded before analysis to ensure that sufficient biological diversity was captured. Alpha diversity and UniFrac[23] analyses were done after subsampling to even depth to reduce bias due to the dependence of these measures on sampling depth. For analyses on phylum and genus level the following steps were taken: 1) Greengenes suggested taxa assignments were heeded (*e*.*g*. [*Prevotella*] was treated as *Prevotella*), 2) spurious taxa with mean abundance under 0.01% were removed, 3) each sample was normalized to relative abundance by dividing by total abundance, and finally 4) unclassified taxa at the given rank were removed from further analysis.

Carriage (presence or absence; *i*.*e*. prevalence) of each genus was compared in three groups, and odds ratios (ORs) were calculated for genus, based on unconditional logistic regression modelling, adjusting for age, sex, education, smoking, alcohol drinking, family history of ESCC, MFT score (the number of missing and filled teeth), times of tooth brushing per day, daily consumption of pickled vegetables and daily consumption of fresh fruits. Relative abundance of each genus was compared using the Metastats package.[24] False discovery rate (FDR) adjustment was used to correct for multiple comparisons. Principal coordinate analysis (PCoA) was applied to ordinate dissimilarity matrices. A multinomial logistic regression model was used to compare the first 10 coordinates from PCoA among groups of study subjects.

### Ethical considerations

The study was approved by the Institutional Review Board of School of Life Sciences, Fudan University and the Institutional Review Board of Qilu Hospital, Shandong University (). Written informed consent was obtained from all participants before interview and sample collection.

## Results

**Fig 1:**
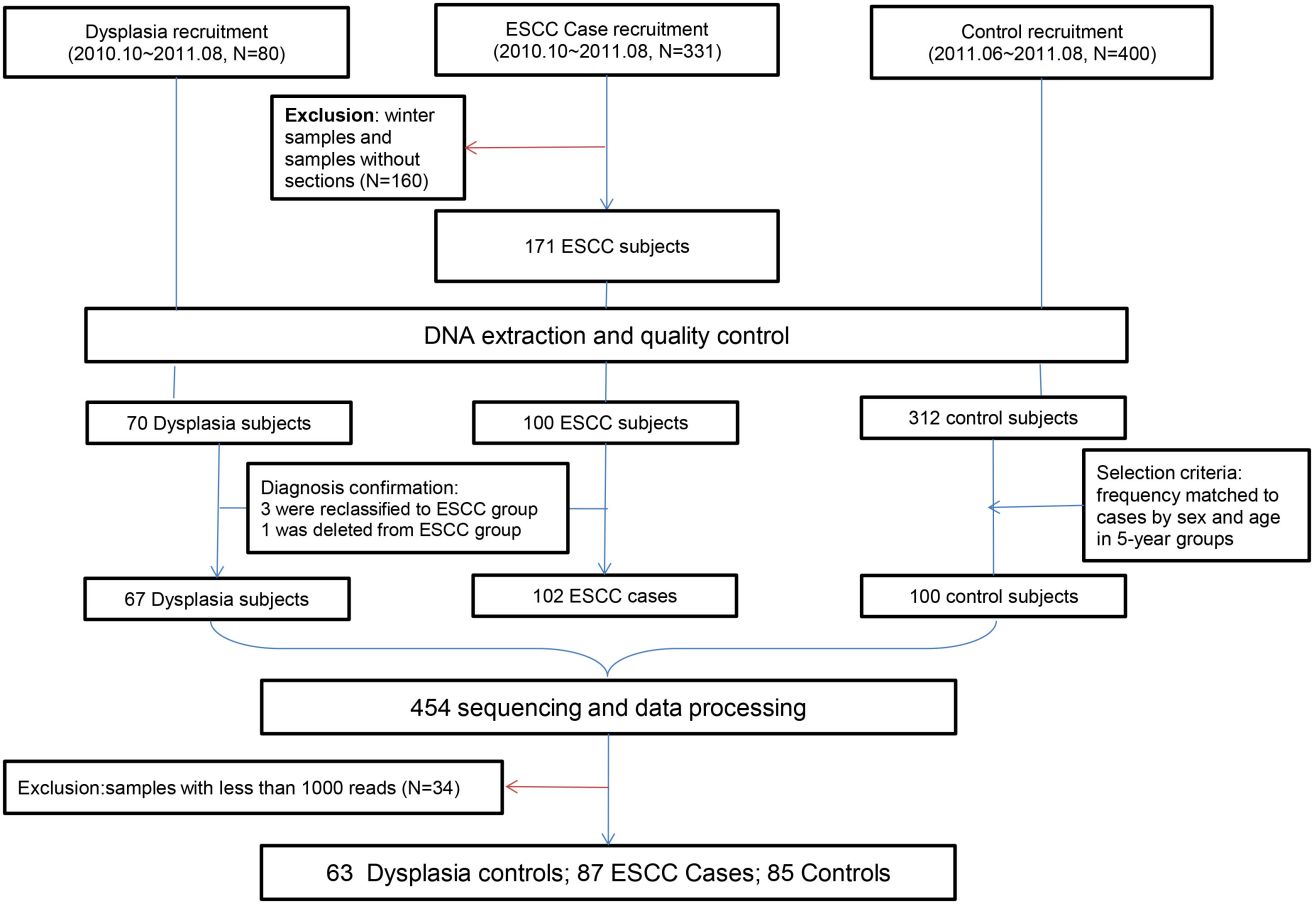
Flowchart of sample selection.

Multiplexed, barcoded sequencing data were deconvoluted and a total of 1.7M amplicon reads was obtained. Thirty-four samples had less than 1000 reads and were excluded (235 samples were left. Fig 1). Approximately 52% of all reads were discarded due to insufficient quality or read length less than 300 bp (384K short reads and 471K low quality reads), leaving 800K reads with 3402 average good quality reads per sample. The final data contained 32,192 unique reads and clustered into 489 OTUs.

Finally, 87 patients with ESCC (ESCC group, 59 males and 28 females), 63 patients with dysplasia (Dysplasia group, 41 males and 22 females), and 85 control subjects (Control group, 62 males and 23 females) remained for further analysis. The clinical parameters including age, sex, education, smoking status, drinking status, MFT score, times of tooth brushing per day, family history of ESCC and daily consumption of pickled vegetables and fruits are shown in Table 1. Times of tooth brushing per day and daily consumption of pickled vegetables were significantly different among three groups (*P*<0.05); ESCC patients consumed more pickled vegetables and brushed teeth less often compared to Dysplasia and control subjects.

**Table 1:** Basic characteristics of subjects in esophageal squamous cell carcinoma (ESCC), dysplasia control and healthy control (Control) groups.

	ESCC (%)	Dysplasia (%)	Control (%)	
	(N = 87)	(N = 63)	(N = 85)	*P* *
Age (years, mean ± SD)	64.8±8.0	65.5±7.6	66.0±7.3	0.56
Sex				
Men	59(67.8)	41(65.1)	62(72.9)	
Women	28(32.2)	22(34.9)	23(27.1)	0.57
Education				
Illiteracy	26(29.9)	19(30.6)	19(22.4)	
1~5 years	39(44.8)	28(45.2)	42(49.4)	
≥5 years	22(25.3)	15(24.2)	24(28.2)	0.79
Smoking				
Never	36(41.4)	22(36.1)	26(30.6)	
Ever	51(58.6)	39(63.9)	59(69.4)	0.34
Alcohol drinking				
Never	37(42.5)	28(45.9)	51(60.0)	
Ever	50(57.5)	33(54.1)	34(40.0)	0.06
Family history of ESCC				
No	56(64.4)	45(71.4)	63(74.1)	
Yes	31(35.6)	18(28.6)	22(25.9)	0.36
MFT^†^				
None	11(12.9)	15(24.6)	17(20.0)	
1~4	27(31.8)	12(19.7)	25(29.4)	
≥ 4	47(55.3)	34(55.7)	43(50.6)	0.29
Times of tooth brushing per day				
<2	73(84.9)	50(82.0)	53(63.9)	
≥2	13(15.1)	11(18.0)	30(36.1)	<0.01
Daily consumption of pickled vegetables				
<10 g	18(22.2)	14(26.4)	37(43.5)	
≥10 g	63(77.8)	39(73.6)	48(56.5)	0.01
Daily consumption of fresh fruits				
<25 g	41(52.6)	32(57.1)	35(42.7)	
≥25 g	37(47.4)	24(42.9)	47(57.3)	0.21

* *P* values were based on Wilcoxon rank-sum test for continuous variables, and chi-squared test for categorical variables (two-sided).

^†^ MFT referred to sum of missing and filled teeth.

The sequencing reads were assigned to 437 OTUs in the ESCC group, 446 OTUs in the Dysplasia group, and 471 OTUs in the Control group. To evaluate the diversity and richness of bacterial types in the samples, Chao1 and Shannon indices were calculated. Observed mean values of Chao1 and Shannon indices were 120.8 and 3.4 for the ESCC group, 129.1 and 3.6 for the Dysplasia group, and 147.2 and 3.7 for the Control group, respectively. Tests for difference in OTU diversity and richness, measured by both mean Chao1 and Shannon indices showed significant differences for ESCC vs Control (*P*<0.001) and ESCC vs Dysplasia (*P*<0.01) (Table 2). At low depth, indices of Chao1, Shannon and mean numbers of OTUs increased sharply in all groups, however, the curves leveled off gradually with the increasing sequencing depth. The differences were always significant among three study groups, even at lower depth (S1 Fig).

**Table 2:** Measurements and comparisons of alpha diversity in esophageal squamous cell carcinoma (ESCC), dysplasia control and healthy control (Control) groups.

Group	OTUs	Mean Shannon	Mean Chao1
ESCC	437	3.4	120.8
Dysplasia	446	3.6	129.1
Control	471	3.7	147.2
*P* value*			
ESCC vs. Control		<0.0001	<0.0001
ESCC vs. Dysplasia		0.0005	0.0869
Dysplasia vs. Control		0.0813	0.0007

* *P* values of pairwise comparisons were based on Wilcoxon rank-sum test. *P* value adjustment method was Holm.

**Fig 2:**
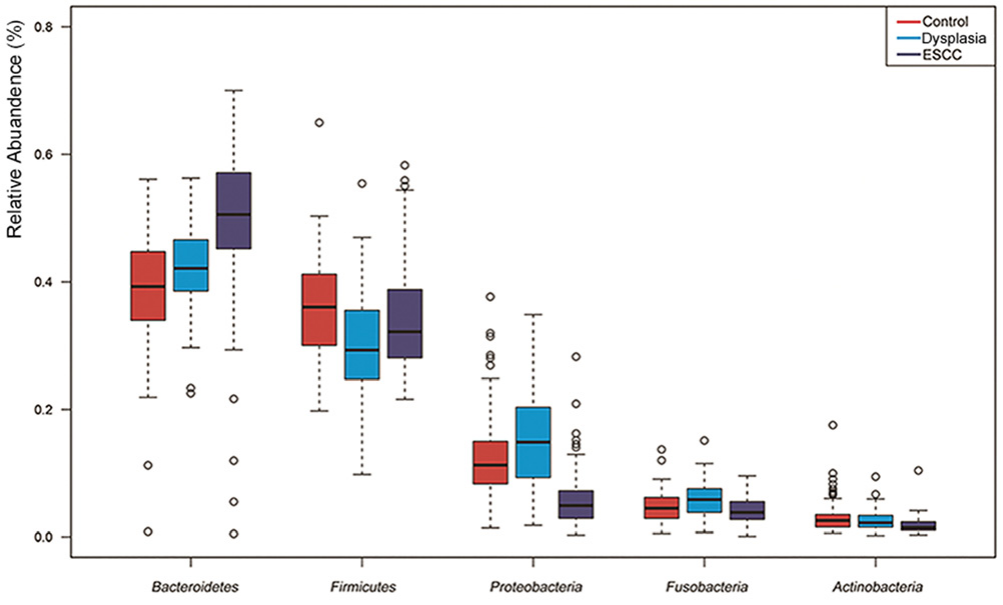
Abundances of microbiota in esophageal squamous cell carcinoma (ESCC), dysplasia control and healthy control (Control) groups at phylum level.

For the overall bacterial community, OTUs were assigned to 12 Phyla and 44 Genera based on the Greengenes database (removing the rare taxa of mean abundance < 0.01%). Phyla composition was consistent across three groups. The five most abundant phyla were the following: *Bacteroidetes* (49.5%, 42.6% and 38.9% in the ESCC, Dysplasia and Control groups, respectively), *Firmicutes* (34.9%, 30.3% and 35.7%), *Proteobacteria* (5.9%, 15.1% and 12.7%), *Fusobacteria* (4.1%, 6.0% and 4.6%) and *Actinobacteria* (1.8%, 2.6% and 3.1%). Fig 2 summarizes the most abundant phyla, representing 80~95% of the bacteria in each sample among the three groups.

In a genus-based analysis, we compared ESCC, dysplasia and control subjects for presence and relative abundance of each specific genus (Table 3). Compared to healthy controls, decreased carriages of several genera in ESCC subjects were found, including *Megasphaera*, *Aggregatibacter*, *Atopobium*, *Lautropia*, *Actinobacillus*, *Bulleidia*, *Catonella*, *Filifactor*, *Corynebacterium*, *TG5*, *Acholeplasma*, *Moryella*, *Butyrivibrio*, *Dialister*, *Peptococcus*, and *Cardiobacterium*. These differences remained for genera, *Lautropia*, *Bulleidia*, *Catonella*, *Corynebacterium*, *Moryella*, *Peptococcus* and *Cardiobacterium*, when comparing ESCC vs. dysplasia subjects. Based on relative abundance, the most predominant genera (>5% in the healthy control group) were *Prevotella* (42.4%, 38.4% and 36.1% in the ESCC, Dysplasia and Control groups, respectively), *Streptococcus* (21.9%, 14.5% and 16.1%), *Veillonella* (7.3%, 10.5% and 13.2%), and *Porphyromonas* (8.9%, 7.0% and 6.5%). Testing for differences in genera abundance revealed an increased level of colonization of members of *Prevotella* (*P*_FDR-adjusted_<0.001), *Streptococcus* (*P*_FDR-adjusted_<0.001) and *Porphyromonas* (*P*_FDR-adjusted_<0.001), and a decreased level of most other genera in the ESCC group compared to non-ESCC groups, although for those genera with a low relative abundance the patterns were not clear.

**Table 3:** Prevalence and relative abundance of genera in esophageal squamous cell carcinoma (ESCC), dysplasia control (Dysplasia) and healthy control (Control) groups.

	% Positive (carriage) *					Abundances % †				
Genus	ESCC	Dysplasia	Control	OR (95%CI) ‡		ESCC	Dysplasia	Control	*P* §	
				ESCC vs. Control	ESCC vs. Dysplasia				ESCC vs. Control	ESCC vs. Dysplasia
*Prevotella*	100	100	100	NA	NA	42.4	38.4	36.1	<0.01	0.04
*Streptococcus*	100	100	100	NA	NA	21.9	14.5	16.1	<0.01	<0.01
*Veillonella*	100	100	100	NA	NA	7.3	10.5	13.2	<0.01	<0.01
*Porphyromonas*	98.9	100	100	NA	NA	8.9	7.0	6.5	<0.01	0.02
*Neisseria*	95.4	98.4	100	NA	NA	2.0	5.1	3.7	<0.01	<0.01
*Fusobacterium*	98.9	96.8	100	NA	NA	2.7	3.8	3.1	0.13	<0.01
*Haemophilus*	96.6	98.4	100	NA	NA	1.6	3.5	2.7	<0.01	<0.01
*Rothia*	97.7	100	100	NA	NA	1.3	1.9	2.4	<0.01	0.01
*Leptotrichia*	100	100	100	NA	NA	1.5	2.6	1.9	0.05	<0.01
*Capnocytophaga*	98.9	100	98.8	NA	NA	2.3	2.5	1.5	<0.01	0.70
*Treponema*	92.0	87.3	97.7	0.30(0.05~1.75)	1.75(0.42~7.32)	0.8	1.0	1.5	<0.01	0.45
*Megasphaera*	82.8	88.9	97.7	0.09(0.02~0.52)¶	0.64(0.21~1.95)	0.4	0.5	1.4	<0.01	0.21
*Campylobacter*	97.7	100	98.8	0.35(0.02~5.25)	NA	0.6	1.2	1.3	<0.01	<0.01
*Selenomonas*	95.4	93.7	100	NA	1.03(0.21~5.01)	0.8	0.7	1.1	0.01	0.70
*Aggregatibacter*	75.9	82.5	94.1	0.18(0.06~0.57)¶	0.61(0.24~1.58)	0.5	1.1	1.0	<0.01	<0.01
*Peptostreptococcus*	92.0	98.4	98.8	0.17(0.02~1.78)	0.43(0.04~4.24)	0.6	0.7	0.9	0.01	0.35
*Oribacterium*	96.6	98.4	98.8	0.45(0.04~5.45)	0.48(0.04~6.35)	0.5	0.8	0.7	<0.01	<0.01
*Actinomyces*	92.0	98.4	96.5	0.38(0.08~1.86)	0.23(0.02~2.24)	0.5	0.7	0.7	0.03	0.11
*Moraxella*	24.1	15.9	21.2	1.48(0.62~3.56)	1.39(0.56~3.47)	0.3	0.1	0.6	0.49	0.44
*Atopobium*	79.3	93.7	90.6	0.27(0.09~0.80)¶	0.38(0.11~1.28)	0.2	0.4	0.4	<0.01	<0.01
*Lautropia*	35.6	63.5	84.7	0.07(0.03~0.17)¶	0.17(0.07~0.41)¶	0.0	0.1	0.4	<0.01	<0.01
*Actinobacillus*	48.3	60.3	71.8	0.42(0.19~0.88)¶	0.54(0.25~1.17)	0.2	0.3	0.4	0.28	0.35
*Bulleidia*	39.1	88.9	96.5	0.02(0.01~0.11)¶	0.10(0.03~0.27)¶	0.1	0.4	0.2	<0.01	<0.01
*Parascardovia*	73.6	82.5	85.9	0.42(0.17~1.06)	0.62(0.24~1.60)	0.2	0.2	0.2	0.08	0.44
*Catonella*	63.2	92.1	94.1	0.14(0.05~0.43)¶	0.16(0.05~0.53)¶	0.1	0.2	0.2	<0.01	<0.01
*Filifactor*	50.6	61.9	80.0	0.22(0.10~0.50)¶	0.59(0.27~1.31)	0.1	0.1	0.2	<0.01	0.01
*Tannerella*	81.6	81.0	82.4	0.71(0.28~1.83)	0.96(0.35~2.61)	0.2	0.2	0.2	0.86	0.96
*Corynebacterium*	32.2	61.9	78.8	0.11(0.05~0.25)¶	0.33(0.15~0.74)¶	0.0	0.2	0.2	<0.01	<0.01
*TG5*	49.4	54.0	72.9	0.33(0.15~0.72)¶	0.95(0.43~2.10)	0.1	0.1	0.2	<0.01	0.46
*Mycoplasma*	70.1	58.7	55.3	2.37(1.10~5.13)	1.96(0.87~4.40)	0.2	0.2	0.1	0.68	0.96
*Elizabethkingia*	78.2	85.7	84.7	0.66(0.27~1.60)	0.54(0.19~1.52)	0.2	0.3	0.1	<0.01	0.96
*Acholeplasma*	12.6	23.8	31.8	0.30(0.12~0.77)¶	0.40(0.15~1.08)	0.0	0.0	0.1	0.04	0.77
*Moryella*	40.2	69.8	71.8	0.18(0.08~0.41)¶	0.18(0.08~0.43)¶	0.1	0.1	0.1	0.18	0.06
*Butyrivibrio*	51.7	47.6	72.9	0.27(0.12~0.63)¶	1.18(0.55~2.52)	0.1	0.1	0.1	0.02	0.96
*Dialister*	25.3	34.9	70.6	0.10(0.04~0.25)¶	0.54(0.23~1.29)	0.0	0.0	0.1	<0.01	0.10
*Streptobacillus*	42.5	30.2	32.9	1.03(0.48~2.22)	1.71(0.75~3.87)	0.1	0.1	0.1	0.34	0.76
*Peptococcus*	28.7	50.8	68.2	0.20(0.09~0.44)¶	0.32(0.14~0.71)¶	0.0	0.1	0.1	<0.01	<0.01
*Abiotrophia*	39.1	33.3	54.1	0.49(0.24~1.02)	1.24(0.56~2.75)	0.1	0.0	0.1	0.22	0.05
*Hylemonella*	20.7	38.1	37.7	0.41(0.18~0.96)	0.43(0.19~1.01)	0.0	0.0	0.1	0.20	0.54
*Lactobacillus*	31.0	27.0	21.2	1.77(0.73~4.30)	0.93(0.39~2.19)	0.1	0.0	0.0	0.28	<0.01
*Bacteroides*	44.8	30.2	51.0	0.54(0.25~1.15)	1.75(0.76~4.01)	1.1	0.1	0.0	0.03	0.09
*Cardiobacterium*	12.6	41.3	37.7	0.21(0.08~0.52)¶	0.23(0.09~0.62)¶	0.0	0.0	0.0	<0.01	<0.01
*Odoribacter*	18.4	31.8	18.8	1.13(0.44~2.90)	0.69(0.28~1.70)	0.0	0.0	0.0	0.73	0.13
*Herbaspirillum*	1.6	34.9	1.2	NA	NA	0.0	0.1	0.0	0.62	<0.01

* Percentage of ESCC and control subjects who carried the specific taxon. NA = not applicable.

^†^ Median relative abundance of the specific taxon in people who carry the taxon.

^‡^ Odds ratios (ORs) and 95% confidence intervals (CIs) were derived from unconditional logistic regression models with non-carriers as the referent, adjusting for sex, age, education, smoking, alcohol drinking, family history of ESCC, MFT, times of tooth brushing per day, daily consumption of pickled vegetables and daily consumption of fresh fruits.

^§^ *P* values were derived from the Metastats package. *P* value adjustment method was FDR (false discovery rate).

^¶^ FDR–adjusted *P* values were *P* less than or equal to 0.05.

**Fig 3:**
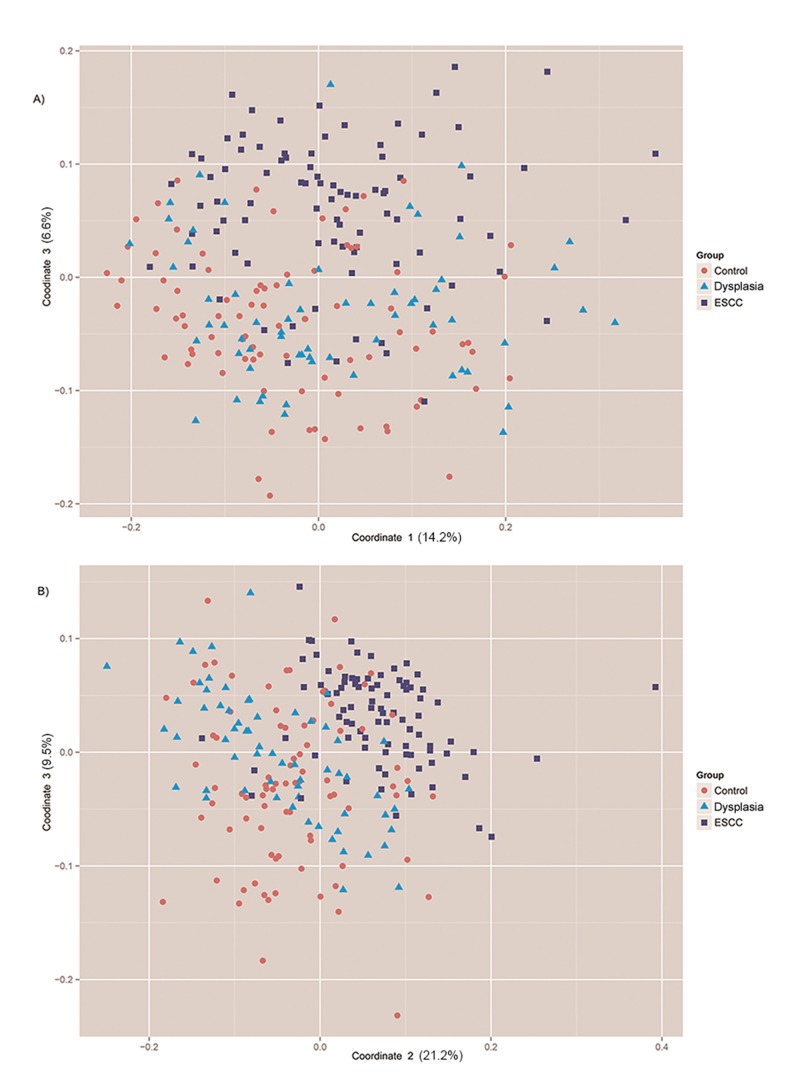
Distribution of samples depicted by the two most significant coordinates for Unifrac (A) and weighted Unifrac distances (B).

We compared the overall bacterial community composition using weighted and unweighted UniFrac distance matrices, and applied PCoA to ordinate the matric. The first 10 coordinates explained 53% and 85% of the variance for Unifrac and weighted Unifrac distances, respectively (S2 Fig). Correlations between coordinates and genera were calculated, and a multinomial logistic regression model was applied to compare the first 10 coordinates in three groups. For Unifrac distance, except for coordinates 7–9, all other coordinates showed significant differences between ESCC and healthy control group, while only coordinates 3, 4, 5 and 8 were significant when comparing ESCC with the Dysplasia group. Results were similar after adjustment for age, sex, education, smoking, alcohol drinking, family history of ESCC, MFT, times of tooth brushing per day, daily consumption of pickled vegetables and daily consumption of fresh fruits (Table 4). For weighted Unifrac distances, highly significant differences (coordinates 2 and 3) emerged when comparing ESCC with both healthy control and Dysplasia groups (Table 4). We further visualized the Unifrac or weighted Unifrac distances using the two most significant coordinates (the ones which explained most variances and with small *P* values) found in multinomial logistic regression analyses (coordinates 1 and 3 for Unifrac distance; coordinates 2 and 3 for weighted Unifrac distance). For both Unifrac and weighted Unifract distances, ESCC and healthy control subjects tended to cluster in opposite directions, while dysplasia subjects were located between the two groups (Fig 3).

**Table 4:** Multinomial logistic regression analysis of the first ten coordinates based on Unifrac and weighted Unifrac distances.

Coordinate	Correlation with genus (Top 5 genera)					ESCC vs.Control		ESCC vs.Dysplasia	
						*P* *	*P* ^†^	*P* *	*P* ^†^
	**Unifrac distance**								
Coordinate 1	*Treponema*	*Filifactor*	*TG5*	*Acholeplasma*	*Aggregatibacter*	0.0016	0.0043	0.3315	0.2289
Coordinate 2	*Lactobacillus*	*Campylobacter*	*Mycoplasma*	*Bulleidia*	*Neisseria*	0.0150	0.0200	0.1313	0.1916
Coordinate 3	*Bulleidia*	*Megasphaera*	*Veillonella*	*Atopobium*	*Porphyromonas*	<0.0001	<0.0001	<0.0001	<0.0001
Coordinate 4	*Prevotella*	*Lautropia*	*Elizabethkingia*	*Haemophilus*	*Actinomyces*	0.0305	0.0384	0.0012	0.0030
Coordinate 5	*Selenomonas*	*Hylemonella*	*Mycoplasma*	*Lactobacillus*	*Filifactor*	0.0133	0.0351	0.0029	0.0044
Coordinate 6	*Capnocytophaga*	*Acholeplasma*	*Treponema*	*Leptotrichia*	*Filifactor*	0.0076	0.0022	0.2457	0.0609
Coordinate 7	*Selenomonas*	*Corynebacterium*	*Leptotrichia*	*Lactobacillus*	*Mycoplasma*	0.0677	0.0135	0.3450	0.3626
Coordinate 8	*Elizabethkingia*	*TG5*	*Streptobacillus*	*Corynebacterium*	*Mycoplasma*	0.1144	0.1451	0.0124	0.0121
Coordinate 9	*TG5*	*Corynebacterium*	*Mycoplasma*	*Catonella*	*Peptostreptococcus*	0.3489	0.7809	0.6814	0.6973
Coordinate 10	*Lautropia*	*Bulleidia*	*TG5*	*Butyrivibrio*	*Abiotrophia*	0.0037	0.0022	0.6490	0.9423
	**Weighted Unifrac distance**								
Coordinate 1	*Prevotella*	*Streptococcus*	*Rothia*	*Peptostreptococcus*	*Haemophilus*	0.9674	0.9863	0.2661	0.2644
Coordinate 2	*Haemophilus*	*Neisseria*	*Prevotella*	*Streptococcus*	*Catonella*	<0.0001	<0.0001	<0.0001	<0.0001
Coordinate 3	*Veillonella*	*Porphyromonas*	*Megasphaera*	*Atopobium*	*Actinomyces*	<0.0001	<0.0001	<0.0001	<0.0001
Coordinate 4	*Fusobacterium*	*Tannerella*	*Treponema*	*Peptococcus*	*Leptotrichia*	0.8960	0.8940	0.0705	0.2545
Coordinate 5	*Streptococcus*	*Leptotrichia*	*Fusobacterium*	*Prevotella*	*Capnocytophaga*	0.0001	0.0003	0.0491	0.0700
Coordinate 6	*Leptotrichia*	*Treponema*	*Streptococcus*	*Campylobacter*	*Dialister*	0.9774	0.8119	0.0361	0.0930
Coordinate 7	*TG5*	*Filifactor*	*Treponema*	*Mycoplasma*	*Bacteroides*	0.0002	0.0001	0.0749	0.0396
Coordinate 8	*Capnocytophaga*	*Streptococcus*	*Filifactor*	*Treponema*	*Dialister*	0.0061	0.0160	0.8769	0.7731
Coordinate 9	*Capnocytophaga*	*Leptotrichia*	*Streptococcus*	*Rothia*	*Neisseria*	0.6133	0.4827	0.4699	0.5216
Coordinate 10	*Peptostreptococcus*	*Atopobium*	*Actinomyces*	*Moryella*	*TG5*	0.2250	0.1265	0.2563	0.1965

* *P* values were derived from Wald chi-squared test, based on unadjusted regression models (10 coordinates were included simultaneously).

^†^ *P* values were derived from Wald chi-squared test. The regression models included 10 coordinates, along with sex, age, education, smoking, alcohol drinking, family history of ESCC, MFT, times of tooth brushing per day, daily consumption of pickled vegetables and daily consumption of fresh fruits.

## Discussion

Increasing evidence indicates a key role for the bacterial microbiota in carcinogenesis.[25] Our study was based on one of the largest sets of 16S rRNA gene sequences from the human oral cavity to evaluate the association between oral microbiota and ESCC risk. We found that ESCC subjects had decreased overall microbial diversity compared to dysplasia and healthy control subjects. In addition, decreased carriage of several genera, including *Lautropia*, *Bulleidia*, *Catonella*, *Corynebacterium*, *Moryella*, *Peptococcus* and *Cardiobacterium*, were found in the ESCC cases compared to non-ESCC subjects. Higher relative abundance of *Prevotella*, *Streptococcus* and *Porphyromonas*, but lower or similar relative abundance of most other genera were observed in ESCC group compared to non-ESCC groups. Multinomial logistic regression analyses on PCoA coordinates of Unifrac or weighted Unifract distances also revealed that ESCC subjects had significantly different levels of several coordinates compared to non-ESCC subjects.

The current study is a sub-study of a case-control study on upper gastrointestinal cancers. For this study, every effort was made to enroll all of the incident ESCC cases in the study area. The frequency matched healthy controls were randomly selected from the general population. The participation rates for both cases and controls were more than 75%. Since we tried to enroll cases before histopathological diagnosis being made, we were also able to include another control group, *i*.*e*. dysplasia patients. The similar directions of associations when using different control groups strengthened the validity of our findings. In addition, histopathological diagnosis of ESCC and dysplasia by a single pathologist, saliva collection after overnight fasting for both cases and controls, and collection of extensive information on potential confounders (*e*.*g*. smoking, alcohol drinking, and other lifestyle factors) were among the strengths of the current study.

Our study also has several limitations. One of the main limitations was that we did not add beads during the process of saliva DNA extraction which might affect the composition and diversity of the oral microbiota. Nevertheless, since the same procedure of saliva DNA preparation was applied in all three groups, the observed differences between groups were still valid, although we could not draw conclusion on those hard-to-break bacteria. As a large fraction of samples did not pass the quanlity control and were excluded, while there might be underlying factors being masked in these subjects, we compared inculed subjects and exclued subjects for the clinical parameters including age, sex, education, smoking status, drinking status, MFT score, times of tooth brushing per day, family history of ESCC and daily consumption of pickled vegetables and fruits, and no significant differences were found. In addition, saliva samples for ESCC cases were collected after endoscopy, which was different from healthy controls. This might raise a concern that the differences of oral microbiota between ESCC cases and healthy controls might be due to contamination during endoscopy. In order to determine whether endoscopy has led (or not) to the changes in the microbiome found in the ESCC population, a small cohort including 30 individuals were enrolled in one of the study hospitals and their saliva samples were obtained both before and after endoscopy. The overall bacterial community composition was analyzed using the same pipeline. No significant difference in bacterial community composition was found between saliva samples collected before and after endoscopy (S3 Fig). Moreover, for subjects in another control group, *i*.*e*. dysplasia subjects, saliva samples were also collected after endoscopy. The similar directions of associations when comparing to different control groups somewhat allayed such a concern. Another limitation is that comparison of the composition and diversity of oral microbiota might be biased by the non-consistent sampling seasons among three groups, even if samples collected in winter in ESCC group were excluded due to the difference of temperature and dietary pattern. However, we compared the composition and diversity between different sampling seasons, but did not find any significant difference between different sampling seasons (data not shown). Finally, due to case-control study design, our results could not distinguish whether decreased microbial richness causes ESCC or is an effect of the cancer status, *e*.*g*. the oral microbiota may be modified by the confunding effect of restricted food intake due to symptoms from extensive lesions and/or dry month in the cancer group. Currently, we are conducting a prospective cohort study, Taizhou Longitudinal Study,[26] in which saliva samples were collected in baseline survey and can be used to prospectively assess the relationship between oral microbiota and the development of ESCC and other gastrointestinal cancers.

In the present study, we found that ESCC subjects had low salivary microbial diversity compared to healthy controls and dysplasia subjects. Similarly, lower bacterial diversity was observed in some other habitats such as the stomach with gastritis[27] and the intestines with colorectal cancer[28]. Most recently, a cross-sectional study in China showed that a decreased microbial richness in the upper digestive tract was associated with cancer-predisposing conditions of the stomach and esophagus (*i*.*e*. low serum pepsinogen I/II ratio and esophageal squamous dysplasia).[29]

Overall, the abundant bacterial groups found in our study are similar to those found in most other studies. The most common phyla in our samples were *Bacteroidetes*, *Firmicutes*, *Proteobacteria*, *Fusobacteria* and *Actinobacteria*. However, our data suggest that the most abundant phylum and genus were *Bacteroidetes* and *Prevotella*, and this might be a little different from other studies which showed *Firmicutes* and *Streptococci* were dominant in oral microbiota.[30] The shift may be due to the different DNA extraction methods and different broad-range PCR primers applied. An alternative explanation for this inconsistency may be the impact of diet and oral hygiene. It has been reported that the composition of oral microbiota is different between rural and urban districts.[31] De Filippo C. *et al*.[32] investigated human intestinal microbiota from children characterized by a modern western diet and a rural diet, and found that children in rural Africa showed a significant enrichment in *Bacteroidetes* and depletion in *Firmicutes*. Individuals with excellent oral hygiene typically harbor a relatively simple flora dominated by gram-positive cocci and rods, mostly comprised of *Streptococci*, however in individuals who do not maintain good oral hygiene, the flora shifts to become more diverse and complex and is dominated by anaerobic gram-negative bacteria, including *Prevotella*.[33] In the present study, even the healthy controls had relatively poor oral hygiene (as indicated by high indices of MFT and few times of tooth brushing per day). All the above reasons may contribute or partly explain the differences of the oral microbiota between different studies.

Our data show that decreased carriage of some genera, *e*.*g*. *Lautropia*, *Bulleidia*, *Catonella*, *Corynebacterium*, *Moryella*, *Peptococcus* and *Cardiobacterium*, are significantly associated with an increased risk of ESCC. However, due to low relative abundances of these genera (<0.5%), there is a danger that the observed differences were due to insufficient sampling depth. On the contrary, presence of genus *Mycoplasma*, which is unaffected by many common antibiotics and reported to be associated with several types of cancer, such as gastric cancer,[34] colon cancer[35] and prostate cancer,[36] was more common in ESCC cases than in healthy and Dysplasia control subjects, although the differences were not statistically significant after FDR adjustment. Higher relative abundance of *Prevotella* and *Streptococcus* were also observed in the ESCC group compared to non-ESCC groups. The proportion of these two genera accounted for nearly 65% of the overall community in ESCC subjects, which might to some extent explain the low diversity of oral microbiota in these patients. Although these genera seem to be non-pathogenic to the host, several studies have indicated that they might be associated with oral and upper digestive tract cancers.[37–39] Further studies are warranted to confirm these findings.

Microbiota and host form a complex “super-organism” in which symbiotic relationships confer benefits to the host in many key aspects of life. A growing body of evidence implicates oral bacteria in the etiology of oral and gastrointestinal cancers.[29, 39] The oral microbiome may play an important role in cancer development, through direct metabolism of chemical carcinogens (e.g. activating alcohol and smoking-related carcinogens locally), and/or through systemic inflammatory effects.[30] Multi-disciplinary collaborations among various fields including epidemiology, microbiology, genetics, immunology, and bioinformatics will be needed to broaden our understanding of the relationship of oral microbiome and cancer risk, and to better understand of cancer etiology.

To summary, this is the first epidemiological study comparing the oral microbiota of ESCC and control subjects while controlling for potential confounders. We observed a correlation between altered salivary bacterial community structure and ESCC risk. The results of our study on the saliva microbiota are of particular interests given its modifiable nature. However, prospective and longitudinal cohort studies are required to verify this finding, along with functional studies, *e*.*g*. metagenomics and transcriptome studies. Establishment of the association between oral microbiome and ESCC risk may lead to significant advances in understanding of cancer etiology, potentially opening a new research paradigm for cancer prevention.

## Supporting Information

S1 Fig Rarefaction curves of indexes in three groups (TIF)

S2 Fig Variations in distance matrix explained by the first 10 coordinates (TIF)

S3 Fig Comparision of the overall bacterial community composition between groups of before and after endoscopy. (TIF)

S1 Checklist STROBE Checklist. (DOCX)

S2 Checklist Clinical Studies Checklist. (DOCX)
