# Supplementary figures and images for: Oral Microbiota and Risk for Esophageal Squamous Cell Carcinoma in a High-Risk Area of China
Source: PLoS One. 2015 Dec 7;10(12):e0143603. doi: 10.1371/journal.pone.0143603 (PMC4671675; doi:10.1371/journal.pone.0143603)

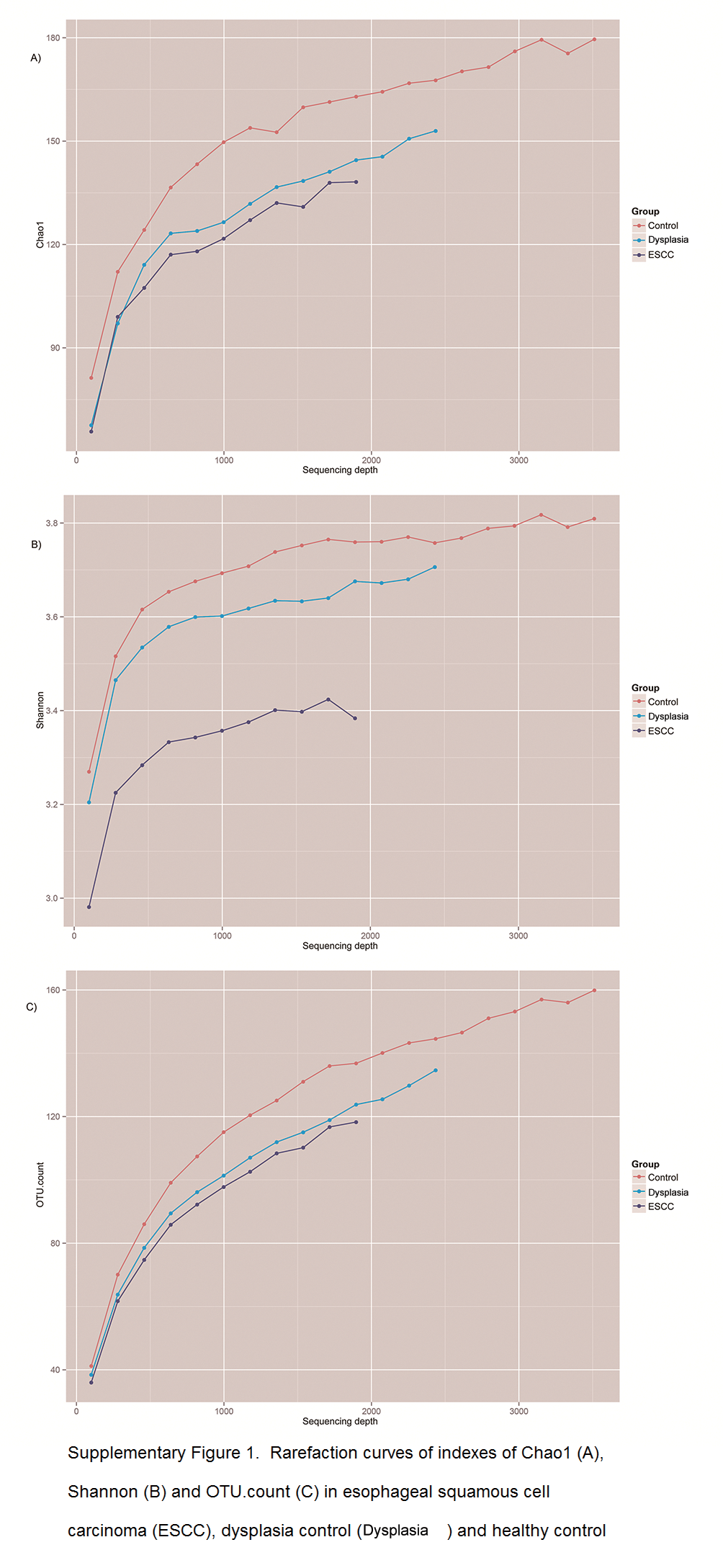

Supplement: S1 Fig — (TIF) [file pone.0143603.s001.tif]

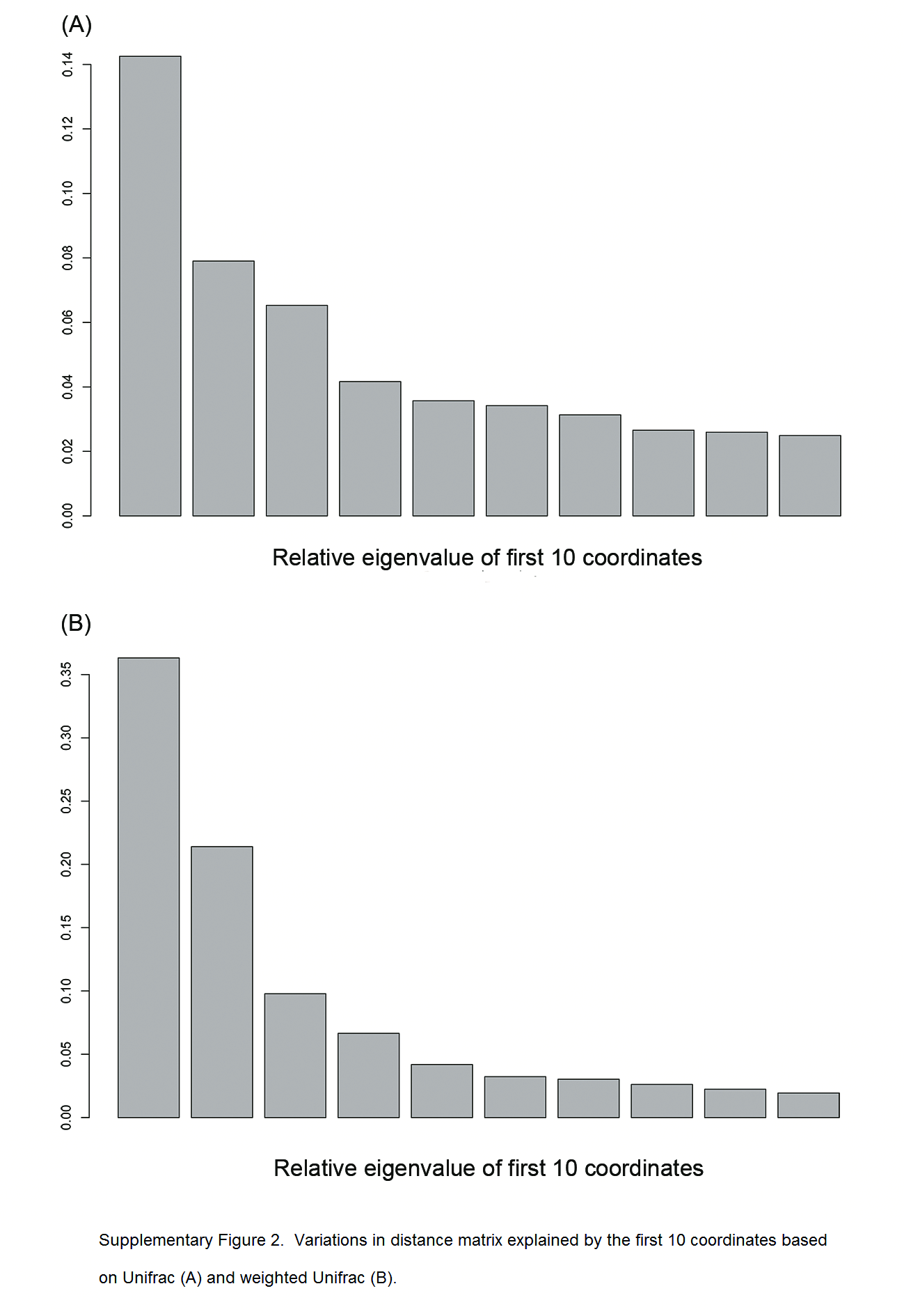

Supplement: S2 Fig — (TIF) [file pone.0143603.s002.tif]

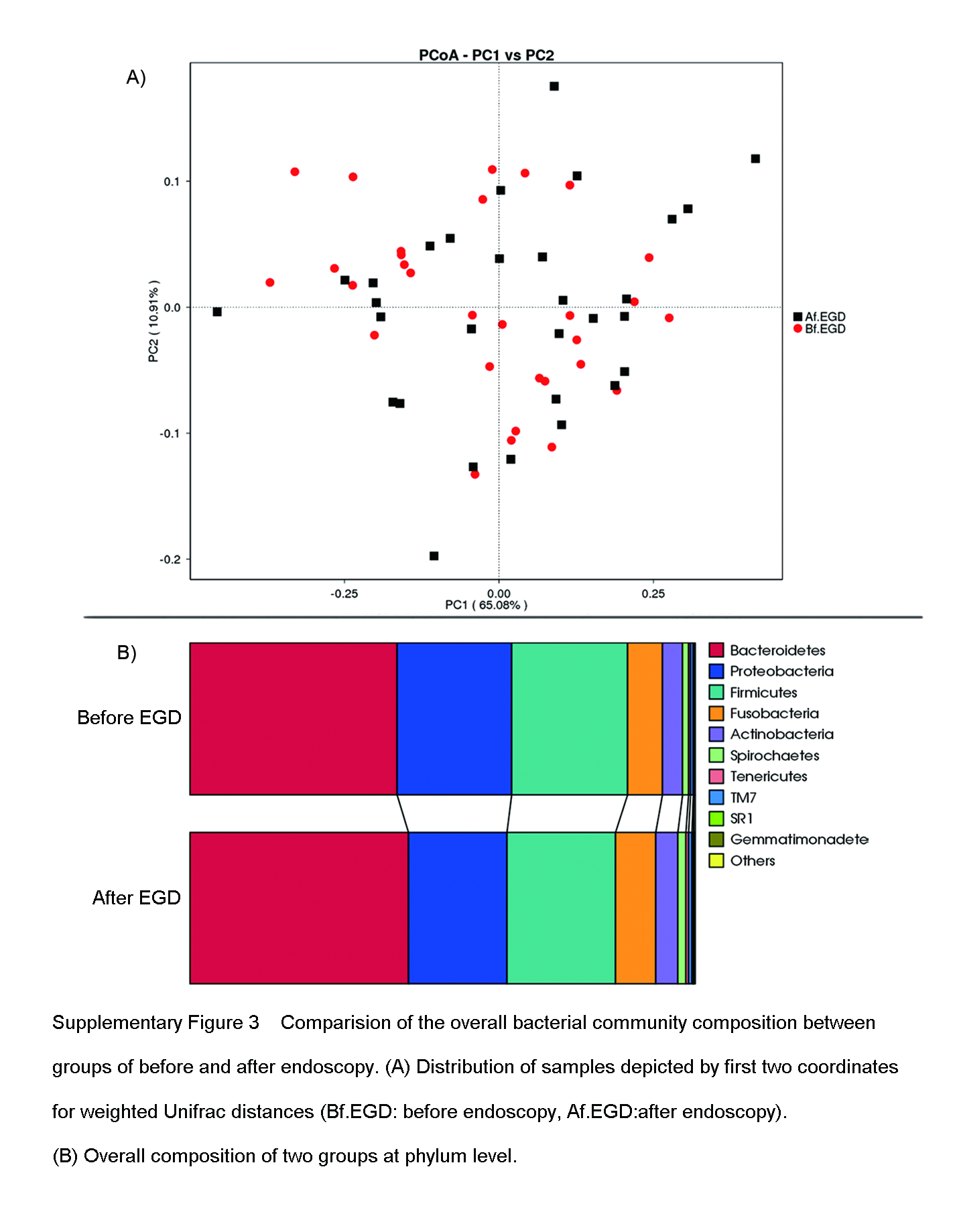

Supplement: S3 Fig — (TIF) [file pone.0143603.s003.tif]
